# Comparative Transcriptomic Profiles of Differentiated Adipocytes Provide Insights into Adipogenesis Mechanisms of Subcutaneous and Intramuscular Fat Tissues in Pigs

**DOI:** 10.3390/cells11030499

**Published:** 2022-01-31

**Authors:** Pan Zhang, Bo Zhang, Peng Shang, Yu Fu, Ruixue Nie, Yangzom Chamba, Hao Zhang

**Affiliations:** 1 National Engineering Laboratory for Animal Breeding, College of Animal Science and Technology, China Agricultural University, Beijing 100193, China; bs20193040382@cau.edu.cn (P.Z.); bozhang0606@cau.edu.cn (B.Z.); B20173040294@cau.edu.cn (Y.F.); s20193040548@cau.edu.cn (R.N.); 2 College of Animal Science, Tibet Agriculture and Animal Husbandry College, Linzhi 860000, China; shangpeng1984@xza.edu.cn (P.S.); yeyourong@xza.edu.cn (Y.C.)

**Keywords:** microRNA, subcutaneous adipocytes, intramuscular adipocytes, RNA-seq

## Abstract

Subcutaneous fat thickness and intramuscular fat content are closely related to meat production and quality in the pig industry. Adipogenesis in adipocytes from subcutaneous and intramuscular fat tissues involves different genes and regulatory mechanisms. Analyzing the data of mRNA and miRNA transcriptomes during the differentiation of adipocytes from these two sources will help identify the different mechanisms of subcutaneous and intramuscular fat deposition. In this study, RNA sequencing technology was used to analyze the differential expression of genes and miRNAs in subcutaneous and intramuscular adipocytes at days 0, 2, 4, and 8 of differentiation. We mainly attributed the difference between fat depositions of the two types of adipocytes to variations in the expression patterns of related genes. Through combined weighted gene co-expression network analysis and K-MEANS, we identified 30 and 22 genes that mainly regulated the differentiation of subcutaneous adipocytes and intramuscular adipocytes, respectively. A total of 17 important candidate miRNAs were identified. This study provides valuable reference for the study of different mechanisms of adipogenesis among subcutaneous and intramuscular fat and contributes to improving pig breeding.

## 1. Introduction

Adipose tissue is one of the largest and most vigorous organs in humans and animals and plays a major role in the control of energy homeostasis [1]. Many human diseases, including diabetes, nonalcoholic fatty liver disease, and cardiovascular disease, are related to lipid deposition and metabolism [2,3]. The formation of adipose tissue mainly comprises two processes: an increase in the number of adipocytes (proliferation) and hypertrophy of adipocytes (differentiation) [4,5]; the latter is accompanied by triglyceride synthesis and storage to form lipid droplets in adipocytes. In pig breeding, subcutaneous fat is reduced to improve the lean percentage and growth rate of the pigs, while intramuscular fat is increased to improve meat quality and flavor [6]. Adipocytes that are distributed intramuscularly and subcutaneously have different developmental processes, compositions, and metabolic functions [7]. The growth rate and lipid content of intramuscular fat are lower than those of subcutaneous fat [8,9]. In vitro experiments on adipogenesis revealed that carbon precursors for fatty acid synthesis in subcutaneous and intramuscular adipocytes are different [10]. Adipogenesis is a complex process that is regulated by various transcription events. In several genes, such as glucose transporters acetyl CoA carboxylase fatty acid synthase and lipoprotein lipase, the different expression levels of these genes may be attributable to the varied mechanisms of lipid formation between the subcutaneous and intramuscular fat tissues [11,12]. During differentiation, primary intramuscular vascular stem cells contain more differentially expressed genes than those in primary subcutaneous vascular stem cells, suggesting that the lipogenic mechanism of these intramuscular cells may be more complicated [13]. A variety of evidence indicates that subcutaneous and intramuscular fats are produced in different ways. However, the different functional genes and regulatory mechanisms of adipogenesis in adipocytes from two sources of subcutaneous and intramuscular fat tissues have not been clarified. 

MicroRNAs (miRNAs) are small non-coding RNAs that regulate the gene expression at the post-transcriptional level and adipogenesis in animals [14,15]. Transcriptomic profiles have been widely used to identify functional genes and non-coding RNAs for phenotype information in animals. Using transcriptomes data from pig backfat tissues and cultured adipocytes, several genes and miRNAs, *SLC27A1*, *ACAA2*, ssc-miR-132, ssc-miR-146b, and ssc-miR-221-5p, were screened that had regulative functions on fat deposition [15,16]. The genes of *POSTN* and *FGFR4* that might regulate intramuscular fat depositin were identified from transcriptomic profiles of intramuscular adipocytes in pigs [17]. The description of the transcriptome landscape comparisons is helpful to understand the regulatory genes on growth rate and meat quality in pigs.

In this study, porcine preadipocytes were isolated from subcutaneous fat and *longissimus dorsi* (LD) tissues and differentiation was induced in vitro. Adipocytes were collected at four time points (0, 2, 4, and 8 days after differentiation) to generate transcriptomic data of mRNAs and miRNAs using RNA-seq. The key functional genes and miRNAs involved in subcutaneous adipocytes or intramuscular adipocytes were identified to understand the regulatory mechanisms of fat deposition in the two tissues of pigs.

## 2. Materials and Methods

### 2.1. Ethics Statement

The animals were reared and handled in accordance with the Guide for the Care and Use of Laboratory Animals in China. All experimental guidelines were approved by the Committee on the Ethics of Animal Experiments of the China Agricultural University (permit number: SKLAB-2012-04-07).

### 2.2. Animals and Isolation of Preadipocytes

Three six-day-old Yorkshire piglets were provided by the Beijing Shunyi Pig Breeding Farm. Intramuscular preadipocytes were isolated from theLD and subcutaneous adipocytes were stripped from the back subcutaneous fat (BF) tissues. First, BF tissues were cut into pieces and digested with 1 mg/mL collagenase type I (Invitrogen, Carlsbad, CA, USA) at 37 °C for 60 min and then filtered through 70 and 200 mesh sieve to remove the undigested part. LD tissues were digested for 1.5–2 h with 2-mg/mL collagenase type I. Preadipocytes were cultured in Dulbecco’s modified Eagle’s medium/Ham’s F-12 (DMEM-F12) growth medium containing 10% fetal bovine serum (FBS) and 1% penicillin–streptomycin at 37 °C in an atmosphere of 5% CO_2_. All culture medium components were obtained from Gibco (Grand Island, NY, USA).

### 2.3. Induced Differentiation of Adipocytes and Oil Red O Staining

When the cell confluency reached 90%, the standard culture medium was replaced with adipogenic induction medium (DMEM-F12 containing 10% FBS, 0.5-mM 3-isobutyl-1-methylxanthine, 1-µM dexamethasone, and 5-µg/mL insulin) (all Sigma, Beijing, China) for two days. Next, the cells were cultured in maintenance medium (growth medium supplemented with 5-μg/mL insulin) for an additional two days. The growth medium was then changed every alternate day until adipocyte maturation.

For Oil Red O staining, the culture medium was removed, and adipocytes were washed thrice with PBS and fixed in 4% formaldehyde for 30 min. Subsequently, 4% formaldehyde was removed, and cells were washed thrice with PBS and stained with Oil Red O for 20 min. Cells were washed thrice with PBS and photographed under a microscope (ZEISS, Jena, Germany). After the oil red O dye was extracted with isopropanol for 20 min, the absorbance was measured at a wavelength of 490 nm to quantify the lipid accumulation.

### 2.4. RNA Extraction, Library Preparation, and Sequencing

Total RNA was isolated from adipocytes after differentiation on days 0, 2, 4, and 8 using the TRIzol reagent (Invitrogen, Carlsbad, CA, USA). RNA concentration and purity were measured using NanoDrop 2000 (Thermo Fisher Scientific, Wilmington, DE, USA). RNA integrity was assessed using the RNA Nano 6000 Assay Kit on the Agilent Bioanalyzer 2100 system (Agilent Technologies, Santa Clara, CA, USA). All RNA samples had RNA integrity number (RIN) values exceeding 8.6. High-throughput sequencing was performed using NovaSeq 6000, and 150 paired-end reads were generated.

For small RNA sequencing, first, 3′ SR and 5′ SR adaptors were ligated, and then reverse transcription of the synthetic first chain was performed. Afterward, PCR amplification was performed, and PAGE gel was used for electrophoresis fragment screening. The rubber was cut and recovered as fragments and purified to obtain a small RNA library. Clustering of the index-coded samples was performed on a cBot Cluster Generation System using the TruSeq PE Cluster Kit v4-cBot-HS (Illumina, New England Biolabs, Ipswich, MA, USA) according to the manufacturer’s instructions. After cluster generation, the library preparations were sequenced on a NovaSeq 6000, and 50 single-end reads were generated.

### 2.5. Read Mapping and Transcriptome Assembly

Clean reads were filtered by removing adapters, reads containing more than 10% unknown nucleotides (N), and low-quality sequences to obtain clean reads. Clean reads were mapped to the pig reference genome (scrofa. Sscrofa11.1, ftp://ftp.ensembl.org/pub/release-99/fasta/sus_scrofa/dna/Sus_scrofa.Sscrofa11.1.dna.toplevel.fa (4 December 2021) using HISAT2(2.0.4) [18,19].

StringTie (1.3.4 d) was used to assemble the transcript and calculate the fragments per kilobase million mapped read (FPKM) values [17]. DESeq2(1.6.3) was used to analyze the differences between the groups [20]. Differentially expressed genes (DEGs) were defined as those with a false discovery rate (FDR) < 0.01 and absolute log_2_ of the fold change |log_2_FC| ≥ 1.

### 2.6. Identification of miRNAs and Prediction of Their Target Genes

Clean reads were mapped to the pig reference genome (scrofa. Sscrofa11.1) with perfect matches using Bowtie software. Annotated tRNA, rRNA, snoRNA, and snRNA sequences were filtered out, and conserved miRNAs were identified using BLAST against miRbase. miRDeep2 software was used to predict new miRNAs based on the characteristics of the miRNA precursor hairpin structure. A differential expression analysis between the two groups was performed using the DESeq2 R package. Differentially expressed miRNAs (DE-miRNAs) were screened with a fold change criterion of  >1.5 and FDR < 0.05 using DESeq2. Target genes of the DE-miRNAs were predicted using miRanda and TargetScan. The miRNA–mRNA signaling pathway interaction network was generated using Cytoscape software (version 3.8.2; San Diego, CA, USA).

### 2.7. Functional Annotation of DEGs and Target Genes of DE-miRNAs

Gene Ontology (GO) enrichment analysis of the DEGs was implemented using the GOseq R packages based on Wallenius noncentral hypergeometric distribution [21], and KOBAS software was used to test the pathway enrichment in the Kyoto Encyclopedia of Genes and Genomes (KEGG).

### 2.8. Weighted Gene Co-Expression Network Analysis

All annotated genes (FPKM > 0.01) were analyzed using the weighted gene co-expression network analysis (WGCNA) package in R studio software. The parameters of the WGCNA program were as follows: minimum module size = 30, minimum height for merging modules = 0.25, and soft threshold = 15. Gene significance (GS) and module membership (MM) refer to the correlation between gene expression and each trait and the correlation between gene expression and each module eigengene, respectively. The correlation between GS and MM was performed using Pearson’s correlation in the WGCNA package. For the genes with eigengene-based connectivity values (|KME|) ≥ 0.7, |MM| ≥ 0.8, and |GS| > 0.3, the pivot gene was selected as the key module.

### 2.9. Verification of DEGs and DE-miRNAs from RNA-Seq

Three genes (*AGT*, *GK*, and *CAT*) and four miRNAs (ssc-miR-874, ssc-miR-370, ssc-miR-210, and ssc-miR-129b) were used for qRT-PCR verification. The primers used for quantitative real-time RT-PCR (qRT-PCR) are presented in Table S1. The FastKingRT Kit (Tiangen Biotech Co., Ltd., Beijing, China) was used to synthesize the first-strand cDNA. qRT-PCR was performed using SuperReal PreMix Plus (SYBR Green) (Tiangen Biotech Co., Ltd., Beijing, China) on a CFX96 Real-Time System (Bio-Rad, Hercules, CA, USA). The gene expression levels were calculated using the 2^−^^ΔΔ^^Ct^ method.

### 2.10. Statistical Analysis

Analysis of the phenotypic differences in the lipid accumulation and gene expression levels was performed using one-way analysis of variance (ANOVA) in SPSS software (version 21.0; IBM Corp. Released 2012. IBM SPSS Statistics for Windows, Armonk, NY, USA). Statistical significance was set at *p* < 0.05, and the results were presented as the mean ± SD or mean ± SE.

## 3. Results

### 3.1. Differentiation Efficiency of Subcutaneous Adipocytes Was Higher Than That of Intramuscular Adipocytes

**Figure 1:**
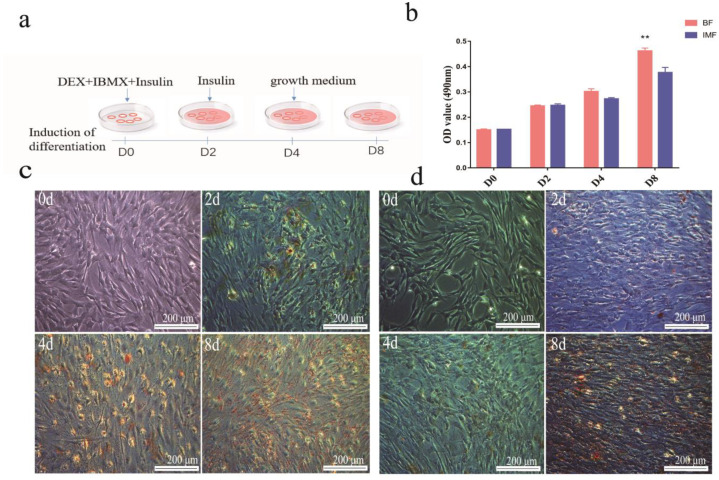
(**a**) Schematic of adipocyte differentiation. (**b**) Absorbance values of destained Oil Red O extracted from the cells at 490 nm. Differentiation of adipocytes from BF and IMF tissues in vitro. (**c**) The four stages of subcutaneous preadipocyte differentiation (days 0, 2, 4, and 8). (**d**) The four stages of intramuscular preadipocyte differentiation. DEX, dexamethasone, IBMX, 3-isobutyl-1-methylxanthine. Data are represented as the mean ± SD, *n* = 3 per group. ** *p* < 0.01 (days 0, 2, 4, and 8). Note: “BF” in the figures and tables in this article represent subcutaneous adipocytes derived from subcutaneous fat on the back, and “IMF” represents intramuscular adipocytes.

The induced differentiation process is illustrated in Figure 1a. After the induction of differentiation, the preadipocytes gradually changed from fibrous to spherical. The subcutaneous preadipocytes began to appear as lipid droplets on the second day of differentiation, and they gradually accumulated as the differentiation time increased; a large number of these droplets were observed on day 8 (Figure 1c).

Compared to subcutaneous preadipocytes, intramuscular preadipocytes slowly produce lipid droplets. Only a small part of these droplets was formed after two days of differentiation, and their amount on the fourth day was similar to that on the second day. However, on the eighth day, the accumulation of lipid droplet accumulation increased significantly (Figure 1d). In general, the total number of lipid droplets produced by intramuscular adipocytes was less than that produced by subcutaneous adipocytes (Figure 1b).

### 3.2. Overview of the Sequencing Data

The transcriptomes of the 24 samples generated 160.63 Gb of clean data, and the percentage of clean Q30 bases ranged from 90.40% to 96.24%. The clean reads of each sample were aligned with the reference genome (Scrofa 11.1), and the mapping ratio ranged from 90.40% to 96.24% (Table S2. For miRNA sequencing, we obtained a total of 327.35 million clean dates, and the Q30 of all samples exceeded 95.13% (Table S2), indicating that the sequencing data can accurately represent the miRNA transcriptome profile in the cell. A total of 25,462 genes and 1587 miRNAs were identified using RNA-seq. The sum of the FPKM value of all samples > 1 was considered to be a reliably expressed gene. Of these, 16,135 genes were reliably expressed (Figure S1a). Of the 1587 miRNAs, 363 were known, and 1224 were newly predicted (Table S3). The known and new miRNAs identified by sequencing and the overall miRNA length distributions were mainly concentrated in 21–23 nt, with the most abundant distribution being 22 nt (Figure S1). 

### 3.3. Differentially Expressed Genes and miRNAs between Stages and between the Two Preadipocytes during Differentiation

**Figure 2:**
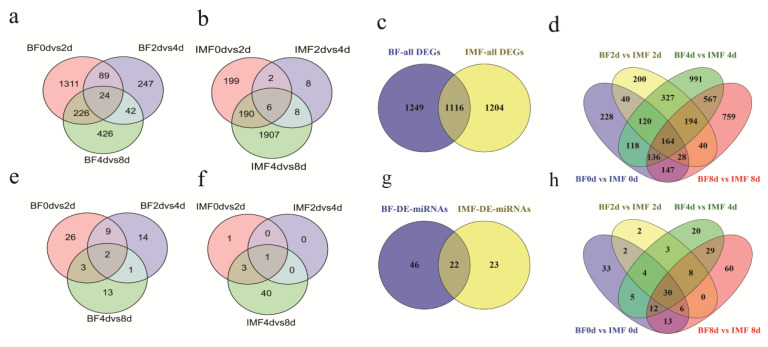
Number of differentially expressed genes (DEGs) and differentially expressed miRNAs (DE-miRNAs). (**a**) Overlapping statistics of DEGs in adjacent stages during the differentiation of subcutaneous adipocytes. (**b**) Overlapping statistics of DEGs in adjacent stages during the differentiation of intramuscular adipocytes. (**c**) Comparison of the total DEGs during the differentiation of subcutaneous adipocytes and total number of DEGs during the differentiation of intramuscular adipocytes. (**d**) Overlapping statistics of DEGs obtained by comparing subcutaneous adipocytes and intramuscular adipocytes at the same differentiation period. (**e**) Overlapping statistics of DE-miRNAs in adjacent stages during the differentiation of subcutaneous adipocytes. (**f**) Overlapping statistics of DE-miRNAs in adjacent stages during the differentiation of intramuscular adipocytes. (**g**) Comparison of the total DE-miRNAs during the differentiation of subcutaneous adipocytes, and the total DEGs during the differentiation of intramuscular adipocytes. (**h**) Overlapping statistics of DE-miRNAs obtained by comparing subcutaneous adipocytes and intramuscular adipocytes at the same differentiation period. Note: “BF” in the figures and tables in this article represents subcutaneous adipocytes derived from subcutaneous fat on the back, and “IMF“ represents intramuscular adipocytes.

The statistics for the number of DEGs and DE-miRNAs are shown in Figure 2. More DEGs and DE-miRNAs in the early stages of subcutaneous adipocyte differentiation were observed than those in the differentiation of intramuscular adipocytes. During the differentiation of subcutaneous adipocytes, the largest number of DEGs appeared in BF-0d vs. BF-2d. In intramuscular adipocytes, IMF-4d vs. IMF-8d exhibited the largest number of DEGs during differentiation, and the number of DE-miRNAs demonstrated the same distribution trend.

A total of 2365 DEGs and 68 DE-miRNAs were found in subcutaneous adipocytes during differentiation, and 24 DEGs and two DE-miRNAs were shared by all three pairs between stages (Figure 2a,e). During the differentiation of intramuscular adipocytes, 2320 DEGs and 45 DE-miRNAs were tested; six DEGs and one DE-miRNA were shared by the three comparison groups (Figure 2b,f).

Upon comparing all DEGs and miRNAs in the differentiation process of the two adipocytes, 1116 overlapping genes and 22 overlapping miRNAs were identified. During the differentiation of subcutaneous adipocytes, 1249 specific DEGs and 46 specific DE-miRNAs were identified, whereas 1204 DEGs and 23 DE-miRNAs were specific differentially expressed in intramuscular adipocytes (Figure 2c,g). Upon comparing the two kinds of adipocytes at the same differentiation stage, a total of 4059 DEGs and 227 DE-miRNAs were identified. Among these, 164 genes and 30 miRNAs were differentially expressed in the four differentiation stages (Figure 2d,h).

### 3.4. Enrichment Analysis of Signal Pathways of DEGs

**Figure 3:**
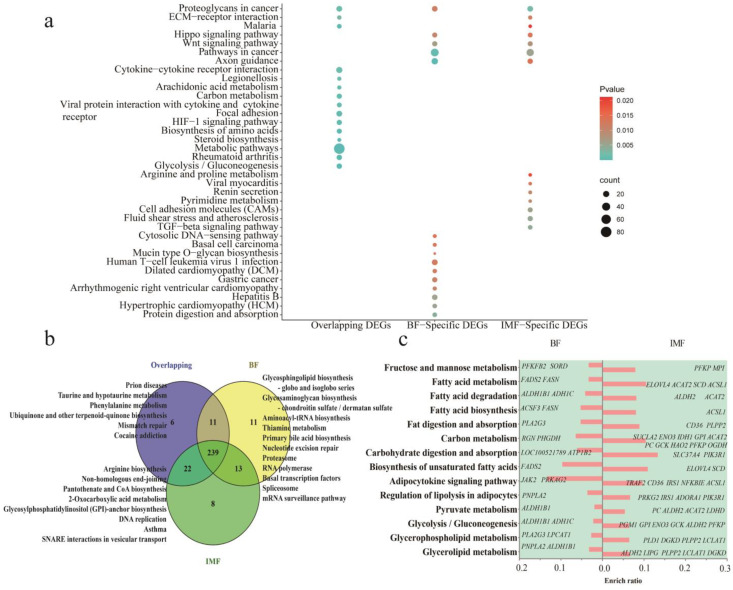
Functional enrichment analysis and statistics of differentially expressed genes (DEGs). (**a**) The top 15 most significant pathways for the enrichment of all specific DEGs and overlapping DEGs during the differentiation of subcutaneous adipocytes and intramuscular adipocytes. (**b**) Statistics of all enrichment pathways. (**c**) Different genes that target the same pathway in the two adipocytes.

We performed a functional enrichment analysis of the overlapping DEGs and specific DEGs in the differentiation process of the two adipocytes. Overlapping DEGs enriched 278 signaling pathways. Subcutaneous and intramuscular adipocyte-specific DEGs were enriched in 274 and 282 pathways, respectively. The main enriched pathways were glycolysis/gluconeogenesis, rheumatoid arthritis, metabolic pathways, steroid biosynthesis, amino acid biosynthesis, HIF-1 signaling pathway, focal adhesion, interaction of viral proteins with cytokines and cytokine receptors, carbon metabolism, fatty acid metabolism, and other pathways (Figure 3a and Table S4). In total, 239 overlapping pathways were identified. Specific pathways are shown in Figure 3b. The genes that targeted the same pathway are shown in Figure 3c. More genes targeted fatty acid metabolism and carbohydrate metabolism-related pathways in intramuscular adipocytes than in subcutaneous adipocytes. The above results indicated that the differentiation process of adipocytes from the two sources was regulated by some of the same genes, as well as by some specific genes, and most of the pathways involved in these genes were the same. 

**Figure 4:**
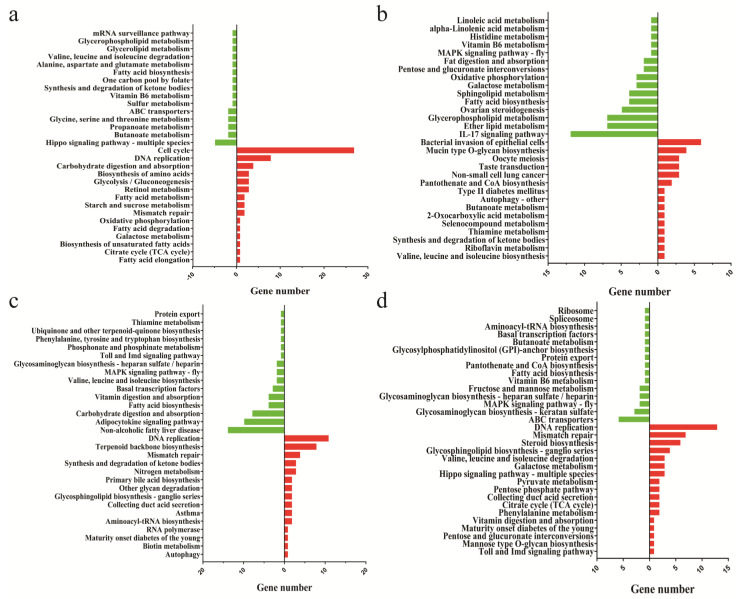
Functional analysis using the Kyoto Encyclopedia of Genes and Genomes (KEGG). (**a**) The KEGG pathway analysis of differentially expressed genes (DEGs) in the BF0d vs. IMF0d group. (**b**) The KEGG pathway analysis of DEGs in the BF2d vs. IMF2d group. (**c**) The KEGG pathway analysis of DEGs in the BF4d vs. IMF4d group. (**d**) The KEGG pathway analysis of DEGs in the BF8d vs. IMF8d group. Red and green represent the pathways enriched by the highly expressed genes of intramuscular adipocytes and subcutaneous adipocytes, respectively.

The DEGs between the two adipocyte groups may explain the differences between the production mechanisms of the two adipocytes. The KEGG analysis showed that, on day 0 of the differentiation, although the induction of differentiation had not yet begun, the highly expressed genes of intramuscular fat cells were enriched in the cell cycle, DNA replication, carbohydrate digestion and absorption, starch and sucrose metabolism, glycolysis/gluconeogenesis, citrate cycle (TCA cycle), and biosynthesis of the unsaturated fatty acids pathway, while the highly expressed genes in subcutaneous fat participate in the Hippo signaling pathway, multiple species, butanoate metabolism, propanoate metabolism, various amino acid metabolism, fatty acid biosynthesis, and other signaling pathways (Figure 4a).

On days 2 and 4 of differentiation, the genes highly expressed in intramuscular adipocytes were mainly involved in DNA replication, synthesis, the degradation of ketone bodies, other glycan degradation, maturity onset diabetes of the young, and other pathways. The highly expressed genes in subcutaneous adipocytes were mainly involved in fatty acid biosynthesis, the adipocytokine signaling pathway, the MAPK signaling pathway, ether lipid metabolism, sphingolipid metabolism, and alpha-linolenic acid metabolism (Figure 4b,c). After eight days of differentiation, genes with a high expression in intramuscular adipocytes were mainly involved in pathways related to glucose metabolism, such as the pentose phosphate pathway, the Toll and Imd signaling pathway, the Hippo signaling pathway, multiple species, phenylalanine metabolism, and nitrogen metabolism. The genes highly expressed in subcutaneous adipocytes are mainly involved in the mRNA surveillance pathway, spliceosome, ribosome, and fatty acid biosynthesis (Figure 4d). 

### 3.5. WGCNA Network Construction

**Figure 5:**
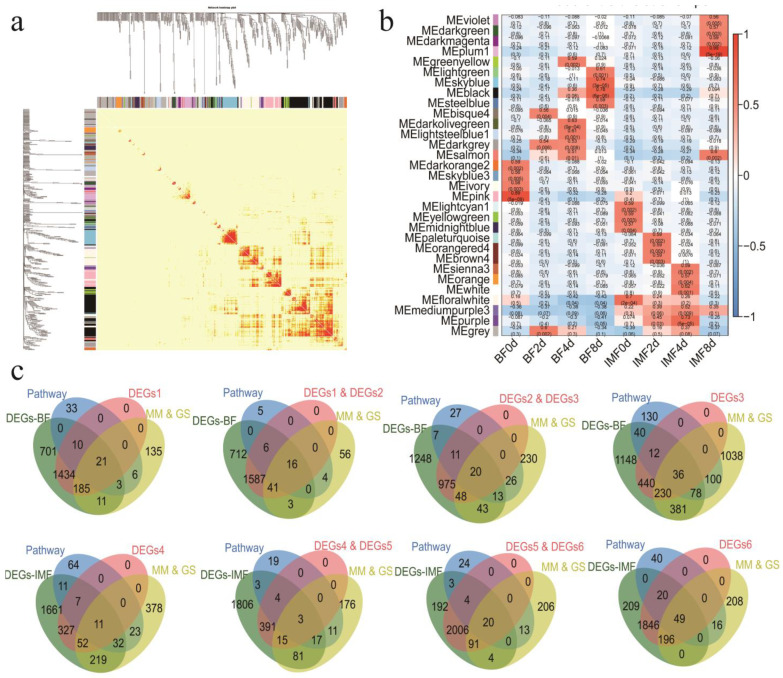
Weighted gene co-expression network analysis (WGCNA). (**a**) Interaction relationship of co-expression genes. (**b**) Heatmap of the correlation between the module eigengenes and differentiation of adipocytes. (**c**) The intersection of the characteristic genes of the module and the differential genes at each stage of differentiation obtains important candidate genes related to the differentiation of adipocytes at each stage of differentiation. The four venn diagram from left to right in the first row represent important genes associated with day 0, 2, 4, and 8 of subcutaneous adipocyte differentiation, respectively. The second row represents the important genes associated with day 0, 2, 4, and 8 of intramuscular adipocyte differentiation, respectively. DEGs1: BF-0d vs. BF-2d; DEGs2: BF-2d vs. BF-4d; DEGs3: BF-4d vs. BF-8d; DEGs4: IMF-0d vs. IMF-2d; DEGs5: IMF-2d vs. IMF-4d; and DEGs6: IMF-4d vs. IMF-8d.

A total of 30 modules were identified. The number of module genes ranged from 41 to 959 (Figure 5a). Analysis of the module–character relationship showed that there were 18 modules related to subcutaneous fat differentiation (*p* < 0.05) and 18 modules for intramuscular fat differentiation (Figure 5b). We performed the KEGG enrichment analysis of the genes in these modules and selected lipid metabolism, carbohydrate metabolism, glycerolipid metabolism, and other pathways that are directly or indirectly related to lipid production. The genes in these pathways served as candidate genes related to the traits in this module.

To further target the candidate genes, we set the relevant module genes in each period as |MM| ≥ 0.8 and |GS| ≥ 0.3 as a more stringent screening criterion. We identified the DEGs that regulate each differentiation stage. A total of 93 DEGs played a regulatory role in the differentiation of subcutaneous adipocytes, of which 21 genes played a role on day 0 of subcutaneous adipocyte differentiation, 16 genes were related to days 2, 20 genes related to day 4, and 36 genes related to day 8. For intramuscular adipocytes, there were 83 genes related to the differentiation process, including 11 related to day 0, 3 related to day 2, 20 related to day 4, and 49 related to day 8 (Figure 5c).

The DEGs between the two adipocytes (IMF vs. BF) in the four periods may be genes that specifically regulate the differentiation of the two adipocytes. We performed the KEGG analysis on the DEGs between the groups and found 466 genes involved in the lipid metabolism-related pathways (Figure S2a and Table S5). 

Combining these 466 genes with genes related to the differentiation of BF and IMF analyzed by the WGCNA, we found 40 genes that may specifically regulate subcutaneous fat deposition, 35 genes that specifically regulate intramuscular fat deposition, and 7 genes that coregulate the differentiation of these two adipocytes (Figure S2b). 

### 3.6. K-Means Co-Expression Trend Analysis of DEGs

**Figure 6:**
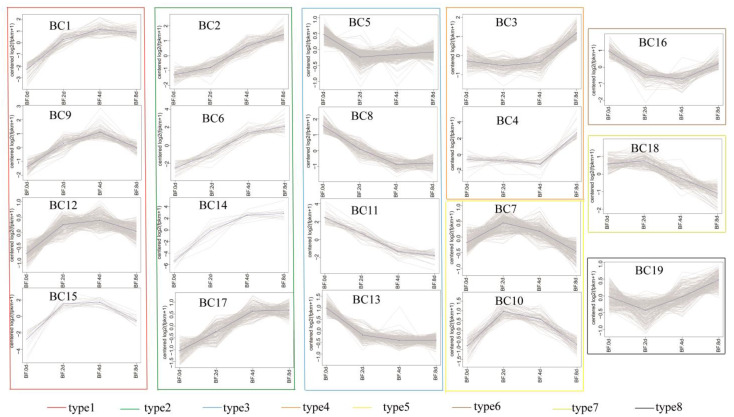
Cluster analysis of the differentially expressed genes (DEGs) expression trends in subcutaneous adipocyte differentiation.

**Figure 7:**
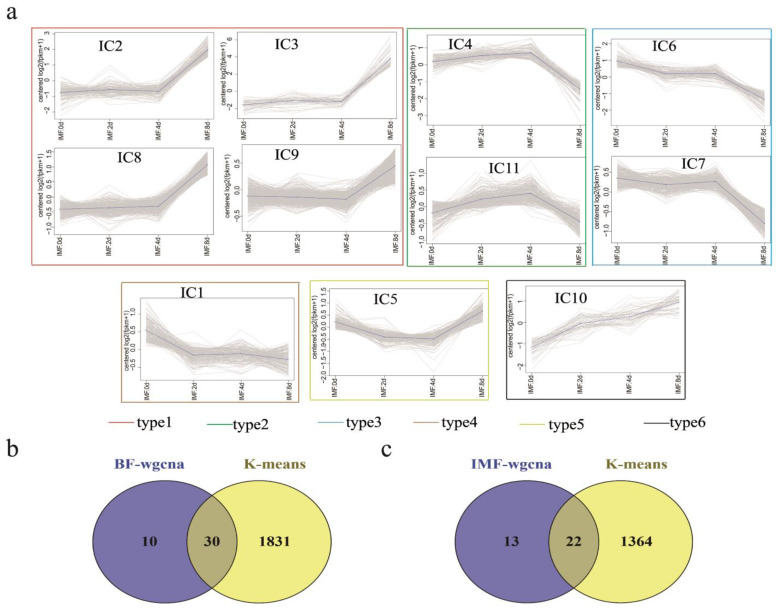
Cluster analysis of differentially expressed genes (DEGs) expression. (**a**) Cluster analysis of DEG expression trends in intramuscular adipocytes differentiation. (**b**) Genes overlapped by the K-means analysis and weighted gene co-expression network analysis (WGCNA) in subcutaneous adipocytes. (**c**) Genes overlapped by the K-means analysis and WGCNA in intramuscular adipocytes.

Genes with similar expression patterns may be subjected to similar regulatory patterns [22]. Therefore, we analyzed the DEGs by comparing their expression trends during adipocyte differentiation. Using K-means clustering for the expression trend analysis, we obtained 19 expression pattern types in the subcutaneous adipocyte group (Figure 6), with the number of genes ranging from 13 to 384 genes. A total of 11 patterns were obtained in the intramuscular adipocyte group, with the number of genes ranging from 51 to 481 (Figure 7a). We merged the modules with the same trend according to the expression trend and, finally, divided them into eight model types in the subcutaneous adipocyte group and six model types in the intramuscular adipocyte component. Next, we combined the specific candidate genes found by WGCNA and the genes obtained by the K-means cluster analysis with 30 genes that may specifically play an important role in the differentiation of subcutaneous adipocytes, including *PCK1*, *STAT1*, *IGF1*, *CYP26B1*, *CAT*, *AGT*, *SOAT1*, *ENO2*, *GK*, and *FABP4* (Figure 7b and Table S6a). For intramuscular adipocytes, 22 genes specifically played an important role in the differentiation process, such as *FGF2*, *COX4I2*, *THSD7A*, *SHC3*, *CACNG7*, and *GLA* (Figure 7c and Table S6b). The expression levels of the 52 genes in the two adipocytes are shown in Figure S3.

### 3.7. miRNA-Target Genes Identification and Functional Annotation

Target genes for all differentially expressed miRNAs (DE-miRNAs) were predicted, and the negative regulatory relationship between the target genes and DEGs was analyzed. A total of 17 DE-miRNAs targeted 187 DEGs in comparison of the adjacent time groups during the differentiation process of the subcutaneous adipocytes, and 12 DE-miRNAs targeted 255 DEGs in intramuscular adipocytes. For group BF0d vs. IMF0d, we found 19 DE-miRNAs negatively regulated 177 DEGs, BF2d vs. IMF2d group, found that three DE-miRNAs negatively regulated four DEGs, the BF4d vs. IMF4d group, found 17 DE-miRNAs negatively regulated 462 DEGs, and for group BF8d vs. IMF8d, 48 DE-miRNAs were found to target 522 genes (Table S7).

The KOBAS online software was used to perform the GO and KEGG functional enrichment analyses of the target genes. In total, 157 KEGG signaling pathways were enriched in 187 genes targeted by 17 DE-miRNAs during subcutaneous adipocyte differentiation. The main pathways included the pentose phosphate pathway, glucagon signaling pathway, fructose and mannose metabolism, Hippo signaling pathway, and propanoate metabolism (Figure S4a). For intramuscular adipocytes, 175 signaling pathways were enriched, and the main pathways included taurine and hypotaurine metabolism, glutathione metabolism, arachidonic acid metabolism, cholesterol metabolism, steroid biosynthesis, purine metabolism, pyrimidine metabolism, fat digestion and absorption, and some disease-related pathways (Figure S4b).

The target gene enrichment analysis of the DE-miRNAs compared between two adipocytes at the same differentiation stage showed that the main enrichment pathways of the target genes of the DE-miRNAs downregulated in intramuscular adipocytes were peroxisome, alanine, aspartate, and glutamate metabolism; fatty acid degradation; valine, leucine, and isoleucine degradation; glycerolipid metabolism; renin secretion; fatty acid metabolism; fluid shear stress and atherosclerosis; and aldosterone synthesis and secretion. The target genes of the DE-miRNAs upregulated in intramuscular adipocytes were mainly enriched for insulin resistance, glycosaminoglycan biosynthesis–keratan sulfate, starch and sucrose metabolism, aldosterone-regulated sodium reabsorption, staphylococcus aureus infection, retinol metabolism, and fructose and mannose metabolism (Figure S4c).

### 3.8. Key miRNAs Identified in Two Adipocytes

**Figure 8:**
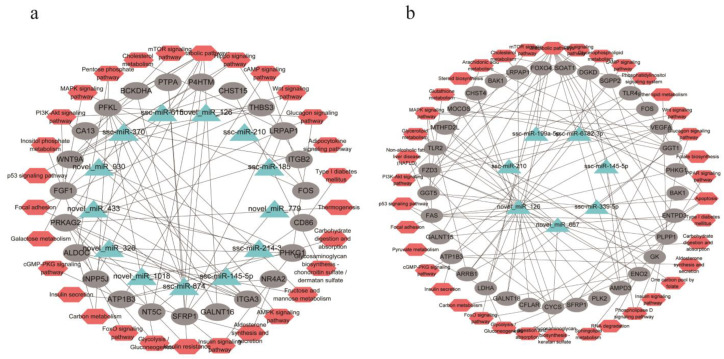
Co-expressed network construction. (**a**) Co-expressed network of differentially expressed miRNAs (DE-miRNAs) from subcutaneous adipocyte and their targeted differentially expressed genes (DEGs) involved in pathways related to fat metabolism and glucose metabolism. (**b**) Co-expressed network of DE-miRNAs from intramuscular adipocytes and their targeted DEGs involved in pathways related to fat metabolism and glucose metabolism. Green represents the differential miRNA, red is the KEGG signaling pathway involved in the target gene, and gray represents the target gene of the miRNA.

To identify the key miRNAs that regulate lipid metabolism, we constructed a co-expression network diagram of miRNAs, targeted mRNAs, and related signaling pathways involved in targeted mRNA. In the subcutaneous adipocyte group, 32 miRNA–mRNA pairs were constructed from 13 miRNAs and 23 targeted mRNAs (Figure 8a). In the intramuscular adipocyte group, 7 miRNAs and 33 targeted mRNAs were used to construct 43 miRNA–mRNA pairs (Figure 8b). Among them, ssc-miR-210, ssc-miR-145-5p, and novel-miR-126 are shown in the two network diagrams, indicating that these three miRNAs play a regulatory role in the two adipocytes. The expression levels of 17 key miRNAs in the differentiation process of the two adipocytes are shown in Figure S5. In addition, ssc-miR-6782-3p was identified, and its expression trend in the two adipocytes was basically the same; therefore, it is speculated to have a regulatory effect on the differentiation of the two adipocytes. In addition to the four aforementioned miRNAs, the remaining miRNAs in the two network diagrams may be important for the two adipocytes. ssc-miR-370 and ssc-miR-874 target a large number of genes related to the adipocytokine signaling pathway, the MAPK signaling pathway, and certain pathways related to glucose metabolism, including *PRKAG2*, *CA13*, *FOS*, *SFRP1*, and *FGF1*. The novel-miR-126 and novel-miR-667 of DE-miRNAs in intramuscular adipocytes target a large number of genes related to glycerolipid metabolism, carbohydrate metabolism, and insulin signaling pathways, such as *ENO2* involved in glycolysis/gluconeogenesis, cholesterol synthesis gene *SOAT1*, lipogenic gene *FAS*, glyceride metabolism gene *GK*, etc. 

### 3.9. Validation of DEGs and DE-miRNAs

**Figure 9:**
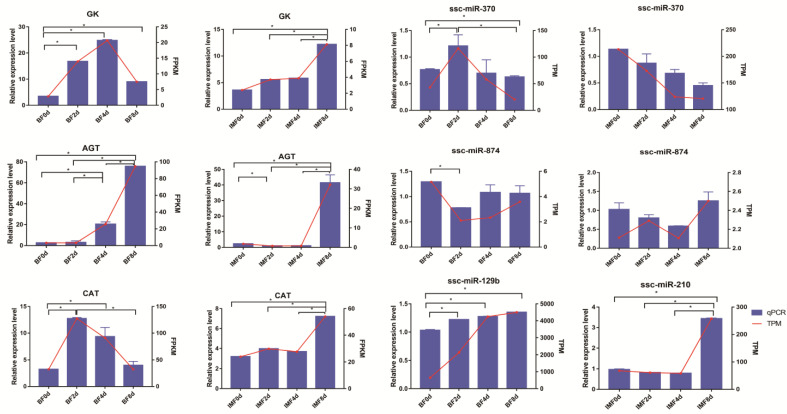
Expression of differentially expressed genes (DEGs) and differentially expressed miRNAs (DE-miRNAs) in the four stages of differentiation of subcutaneous adipocyte and intramuscular adipocyte was verified by qRT-PCR. Data from qRT-PCR are presented as the column and *Y*-axis on the left, while the data from RNA-Seq are presented as the line and *Y*-axis on the right. Data are represented as the mean ± SE, *n* = 3 per group. * *p* < 0.05.

A total of seven genes, including three mRNAs (*AGT*, *GK*, and *CAT*) and four miRNAs (ssc-miR-874, ssc-miR-370, ssc-miR-210, and ssc-miR-129b), were selected for qRT-PCR to validate their expression differences in RNA-Seq. After comparison with the RNA-Seq data, it was found that the expression trend of qRT-PCR was similar to that of RNA-Seq (Figure 9). These results indicate that the RNA-Seq results were reliable and efficient.

## 4. Discussion

Generally, the relative speed of fatty acid synthesis and decomposition determines triglyceride content, which ultimately affects the amount of fat deposition. We tested these genes in subcutaneous and intramuscular adipocytes at the same differentiation time. We found that the highly expressed genes in subcutaneous adipocytes were mainly involved in fatty acid biosynthesis, amino acid metabolism, sphingolipid metabolism, and other pathways. In intramuscular adipocytes, highly expressed genes are mainly enriched in pathways, such as carbohydrate digestion and absorption, starch and sucrose metabolism, glycolysis/gluconeogenesis, and the citrate cycle (TCA cycle). This is consistent with the phenotype where subcutaneous adipocytes accumulate more lipid droplets than intramuscular adipocytes. This also confirms that fats accumulated by the two adipocytes have different carbon precursors [7,23].

The gene expression profile of subcutaneous adipocytes and the intramuscular adipocyte differentiation process provides a lot of information for studying the regulatory mechanism of specific fat depositions in the differentiation of different adipocytes in pigs. We identified 30 genes in subcutaneous adipocytes that may have a specific effect on the differentiation of Yorkshire subcutaneous adipocytes. Half of the genes (*GK*, *CAT*, *FABP4*, *AGT*, *PCK1*, *STAT1*, *IGF1*, *PDE3B*, *APOA1*, *SOAT1*, *CYP26B1*, *ENPP2*, and *HGF*) have been reported to play a role during adipogenesis or in differentiated adipocytes [24,25,26,27,28,29], except for *GGT5*, *LOC100521789*, *LPCAT2*, *PTPR*, *HHAT*, *GADD45G*, *PRKG1*, *ENSSSCG00000035293*, *GRIN3A*, *JAK2*, *CCDC180*, *NFATC1*, *CCBN2*, *CCBN3*, *ENO2*, *PIK3C2B*, and *ADH1C.* When adipocytes are induced to differentiate, preadipocytes re-enter the cell cycle synchronously and undergo mitotic clonal expansion [30]. The differential expression of cyclin B2 (*CCBN2*) and cyclin B3 (*CCBN3*) in subcutaneous adipocytes confirmed this. The expression of these genes changes significantly during the differentiation of subcutaneous adipocytes, and expression levels of most of them exceeded those of intramuscular adipocytes, indicating that these genes may be related to fat deposition in the subcutaneous adipocytes of pigs.

We identified 22 genes related to intramuscular adipocyte differentiation: *ADORA1*, *RAPGEF4*, and *ATP1B1* in the cAMP signaling pathway; *PLPP1* and *ST3GAL5* are involved in glycerophospholipid metabolism; *LDHD* in pyruvate metabolism; *CYP2B22* in retinol metabolism; *SHC3* and *PPP1R3C* in insulin signaling; and the genes related to the proliferation, migration, and differentiation of myoblasts and fibroblasts, such as *ANGPT1* [31], *PRR5L* [32], *PLK1* [33], *PLK2* [34], and *FGF2*, as well as *COX4I2*, *ENSSSCG00000037487*, *CACNG7*, *THSD7A*, *SGPP2*, and *GLA*, whose functions are not yet clear. The biphasic effect of FGF2 in mouse adipocytes is related to its concentration [35] and plays an important role in the differentiation of human preadipocytes [36]. Adiponectin is involved in insulin sensitization, fat metabolism, immunity, and inflammation [37], and *ADORA1* is responsible for anti-lipolysis [38]. Studies have reported that it is a candidate gene that affects the intramuscular fat content [39]. *GNAS* plays multiple important roles in cell fate determination and differentiation during osteogenic differentiation [40]. *NDUFA4L2* is considered to be a gene related to meat quality [41], and *PPP1R3C* is considered to be a candidate gene for intramuscular fat deposition in ducks [42].

miRNAs are small non-coding RNAs that mainly target the 3′ untranslated region of mRNAs to reduce the expression of encoded proteins [43]. Certain studies have used high-throughput sequencing technology to continuously mine miRNAs that play a role in animal adipose tissue and explore their functions [44]. In this study, the combined analysis of DE-miRNAs and DEGs showed that ssc-miR-874 and ssc-miR-370 are important miRNAs for the regulation of pig subcutaneous adipocytes, because they target a large number of genes that are directly or indirectly involved in the regulation of fat metabolism. Fibroblast growth factor (*FGF*) is a pleiotropic growth factor that controls cell proliferation, migration, and differentiation [45]. The results of this study showed that the *FGF1* gene promoter region combined with the *PPAR*γ gene plays a role in regulating adipogenesis. The PPARγ–FGF1 axis, a key regulator of adipose tissue remodeling and systemic metabolic homeostasis, is functionally conserved in mammals [46,47]. In our study, both ssc-miR-370 and ssc-miR-214-3p targeted the *FGF* gene. The ssc-miR-874-, novel-miR-779, and novel-miR-126 genes targeting *SFRP1* are endogenous regulators of Wnt/β-catenin signaling and participate in the paracrine regulation of human adipogenesis [48]. Loss of *Sfrp1* aggravates weight gain, glucose homeostasis, and inflammation in mice with diet-induced obesity [49]. In addition to the genes listed above, ssc-miR-874 and ssc-miR-370 target *HHAT* and *GRIN3A*, respectively, which are considered to be important candidates for the regulation of porcine subcutaneous adipocyte differentiation.

In intramuscular adipocytes, we identified two new miRNAs (novel-miR-667 and novel-miR-126) through the function of target genes. In addition to the two newly identified miRNAs, ssc-miR-6782-3p-*ARRB1* and ssc-miR-199a-5p-*FOXO4* are important miRNA–mRNA pairs that regulate intramuscular fat. The transgene overexpression of *ARRB1* inhibited diet-induced obesity and improved glucose tolerance and systemic insulin sensitivity [50]. Forkhead box O (*FOXO*) proteins are a family of transcription factors, and *FOXO4* is a member of this family. It inhibits the biosynthesis of cholesterol by inhibiting the expression of *CYP51*, leading to the accumulation of dihydrolanosterol [51], and has also been shown to be related to glucose clearance [52].

In conclusion, our results demonstrated that intramuscular and subcutaneous adipocytes had different ways of generating lipid droplets, i.e., different carbon sources. The DEGs and DE-miRNAs in the differentiation process were not the same. We identified 30 genes that play key roles in the differentiation process of subcutaneous adipocytes. Using the constructed miRNA–mRNA signaling pathway network diagram, 10 miRNAs, novel-miR-1018, novel-miR-326, novel-miR-433, novel-miR-930, ssc-miR-185, ssc-miR-370, ssc-miR-874, ssc-miR-615, novel-miR-779, and ssc-miR-214-3p, were found to regulate the differentiation process of subcutaneous adipocytes. In intramuscular adipocytes, a total of 22 genes that may specifically regulate the differentiation process of intramuscular adipocytes were observed. Using the constructed miRNA–mRNA signaling pathway network diagram, ssc-miR-339-5p, ssc-miR-199a-5p, and novel-miR-667, three miRNAs that may regulate the differentiation process of intramuscular adipocytes, were identified.

There is a limitation in the research that needs to be explained. We identified important mRNA–miRNA pairs by predicting the targeting relationship between candidate genes and miRNAs. However, we have not verified their targeting relationship, and future research should focus on the verification of these results.

## Data Availability

All RNA-Seq data were deposited in Gene Expression Omnibusunder, accession number GSE193609 (https://www.ncbi.nlm.nih.gov/geo/query/acc.cgi?acc=GSE193609) (3 January 2022).

## Supplementary Materials

The following supporting information can be downloaded at https://www.mdpi.com/article/10.3390/cells11030499/s1: Figure S1: Length distribution of miRNA. (a) Length distribution of known miRNA. (b) Length distribution of novel miRNA. Figure S2: Differential candidate gene screening. (a) Summary of genes related to fat metabolism in the back subcutaneous fat (BF) vs. intramuscular fat (IMF) groups (0, 2, 4, and 8d). (b) Venn diagram shows the overlapping genes between the lipid metabolism pathway genes of BF vs. IMF and the candidate genes of subcutaneous fat and intramuscular fat in the weighted gene co-expression network analysis (WGCNA). Figure S3: Expression of the key genes during the differentiation of two kinds of adipocytes. (a) Expression of 30 important genes that regulate the differentiation of subcutaneous adipocytes. (b) Expression of 22 important genes that regulate the differentiation of intramuscular adipocytes. The orange broken line indicates the expression of the genes in subcutaneous adipocytes, and the blue indicates the expression of intramuscular adipocytes. Figure S4: Kyoto Encyclopedia of Genes and Genomes (KEGG) analysis of the target genes. (a) KEGG pathway analysis of differentially expressed miRNA (DE-miRNA) target genes from the adjacent differentiation time of subcutaneous adipocytes. (b) KEGG pathway analysis of DE-miRNA target genes from the adjacent differentiation time of intramuscular adipocytes. (c) Pathway enrichment of DE-miRNA target genes in the four stages in the BF vs. IMF. Figure S5: The expression of 17 key miRNA during the differentiation of two kinds of adipocytes. The orange broken line indicates the expression of the miRNA in subcutaneous adipocytes, and the blue indicates the expression of intramuscular adipocytes. Table S1: Primer sequence of qRT-PCR. Table S2: Statistics of transcriptome sequencing data. Table S3: All miRNA expressions. Table S4: Summary of the Kyoto Encyclopedia of Genes and Genomes (KEGG) enrichment pathways. Table S5: Summary of the lipid metabolism-related pathways. Table S6: List of 30 genes that specifically regulate the differentiation of subcutaneous adipocytes, and 22 genes that specifically regulate the differentiation of intramuscular adipocytes. Table S7: Summary of the target genes of different miRNAs in each group.
