# Supplementary material for: Comparative Transcriptomic Profiles of Differentiated Adipocytes Provide Insights into Adipogenesis Mechanisms of Subcutaneous and Intramuscular Fat Tissues in Pigs
Source: Cells. 2022 Jan 31;11(3):499. doi: 10.3390/cells11030499 (PMC8834144; doi:10.3390/cells11030499)
Supplement: Supplementary file 1 [file cells-11-00499-s001.zip › supplementary files/FIgure supplementary.pdf]

Supplementary Figures:

a b

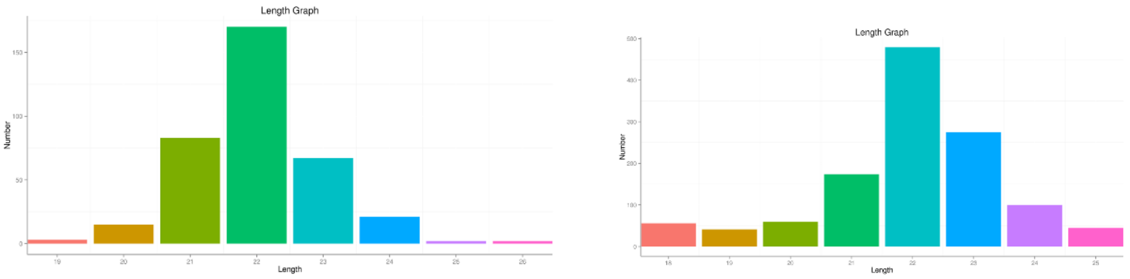

Figure S1. Length distribution of miRNA. (**a**) Length distribution of known miRNA. (**b**) Length distribution of novel miRNA.

a b

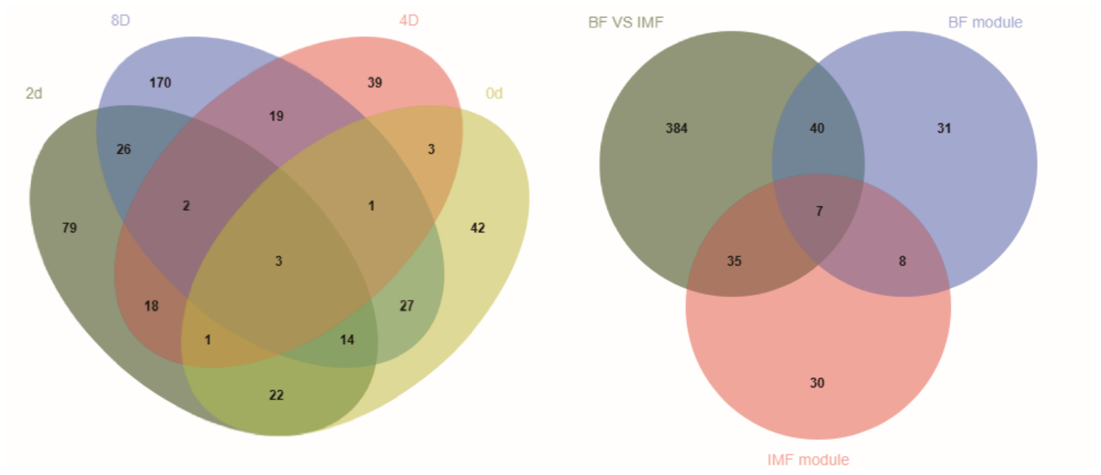

Figure S2. Differential candidate gene screening. (**a**) Summary of genes related to fat metabolism in the back subcutaneous fat (BF) vs intramuscular fat (IMF) group (0 , 2, 4, and 8d). (**b**) Venn diagram shows the overlap genes between the lipid metabolism pathway genes of BF vs IMF and the candidate genes of subcutaneous fat and intramuscular fat in weighted gene co-expression network analysis (WGCNA).

a

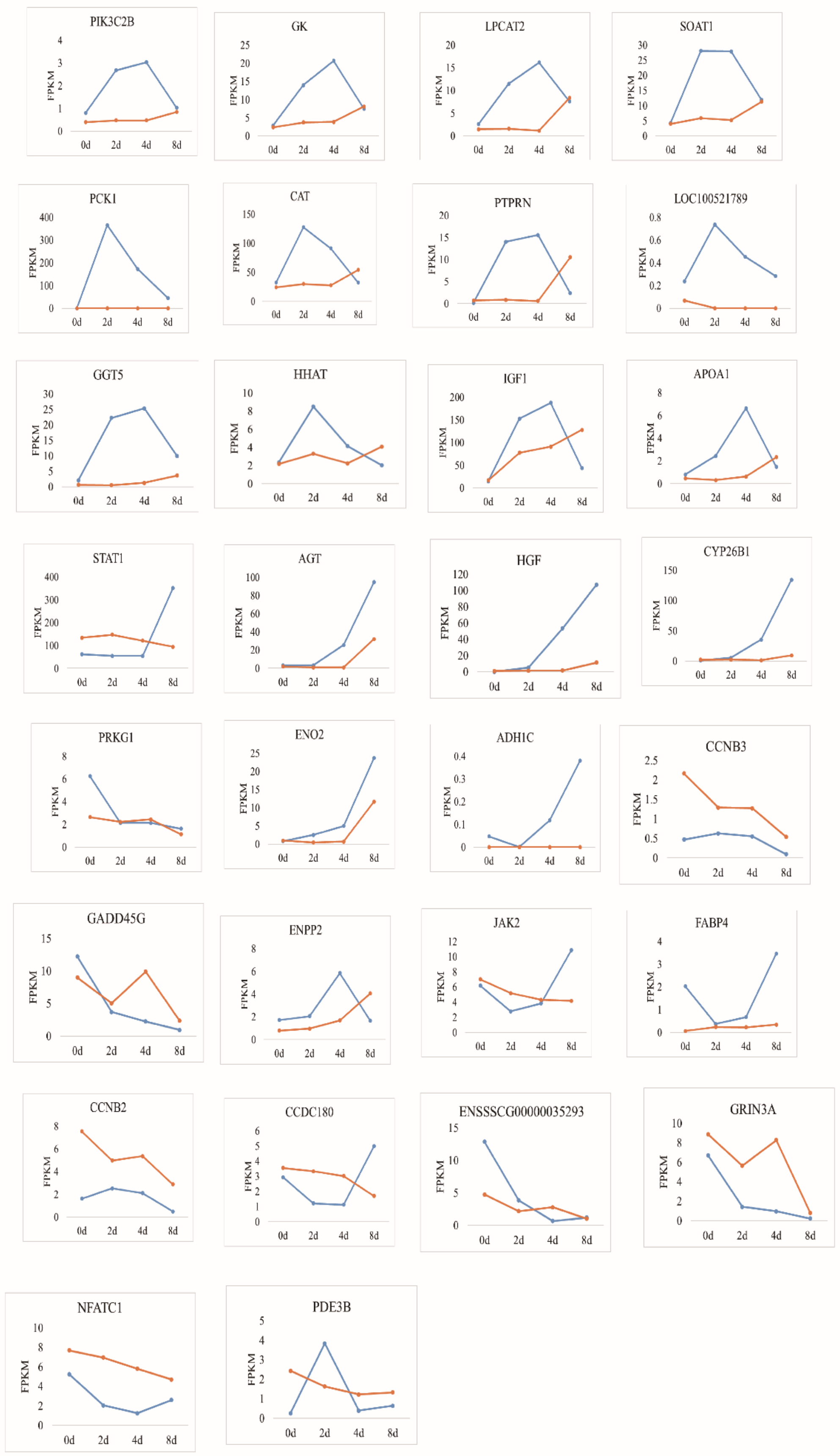
PIK3C2B
GK
LPCAT2
SOAT1
PCK1
CAT
PTPRN
LOC100521789
GGT5
HHAT
IGF1
APOA1
STAT1
AGT
HGF
CYP26B1
PRKG1
ENO2
ADH1C
CCNB3
GADD45G
ENPP2
JAK2
FABP4
CCNB2
CCDC180
ENSSSCG00000035293
GRIN3A
NFATC1
PDE3B
FPKM
0d
2d
4d
8d

b

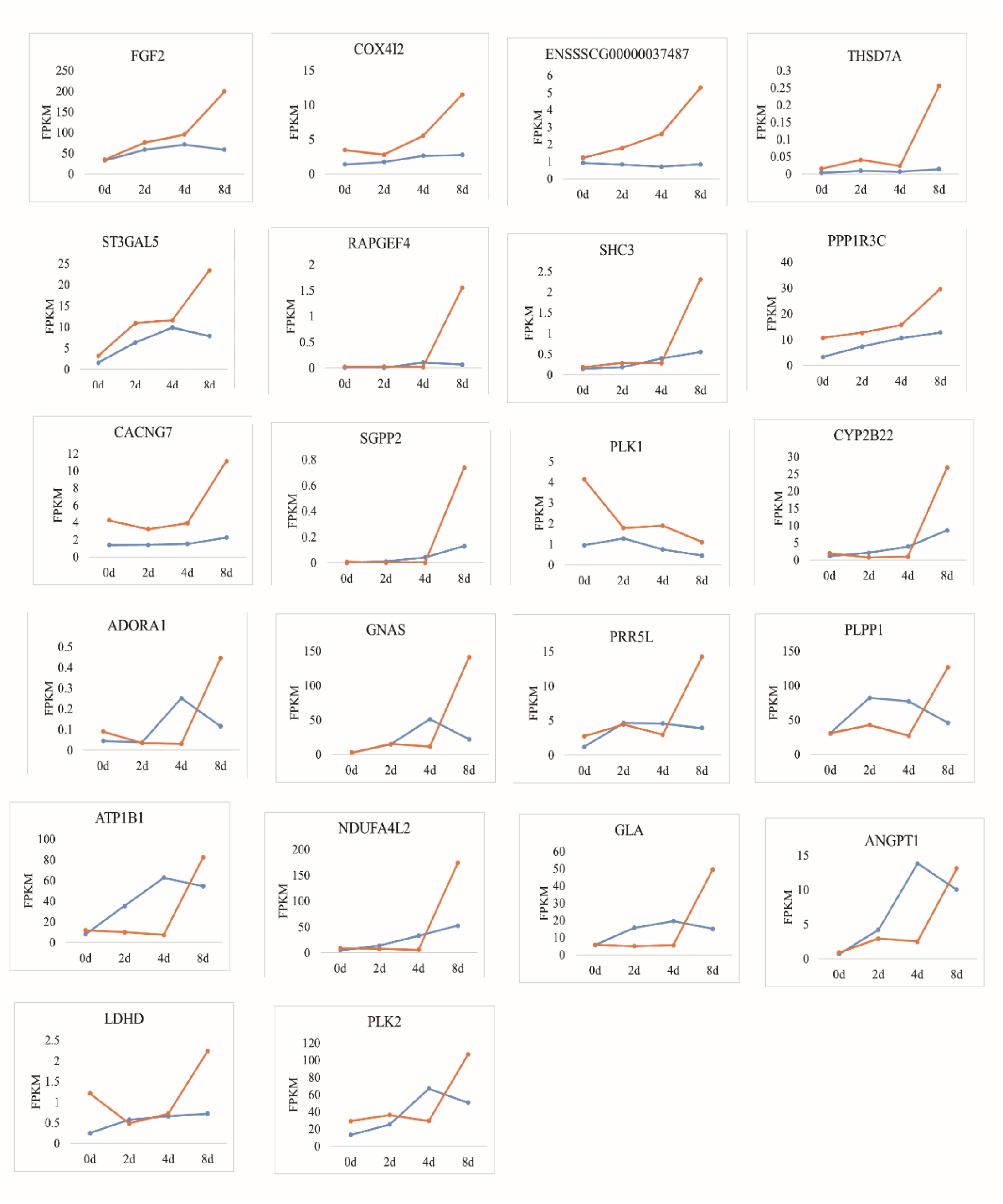

Figure S3. Expression of key genes during the differentiation of two kinds of adipocytes. (**a**) Expression of 30 important genes that regulate the differentiation of subcutaneous adipocytes. (**b**) Expression of 22 important genes that regulate the differentiation of intramuscular adipocytes. The orange broken line indicates the expression of the gene in subcutaneous adipocytes, and the blue indicates the expression of intramuscular adipocytes.

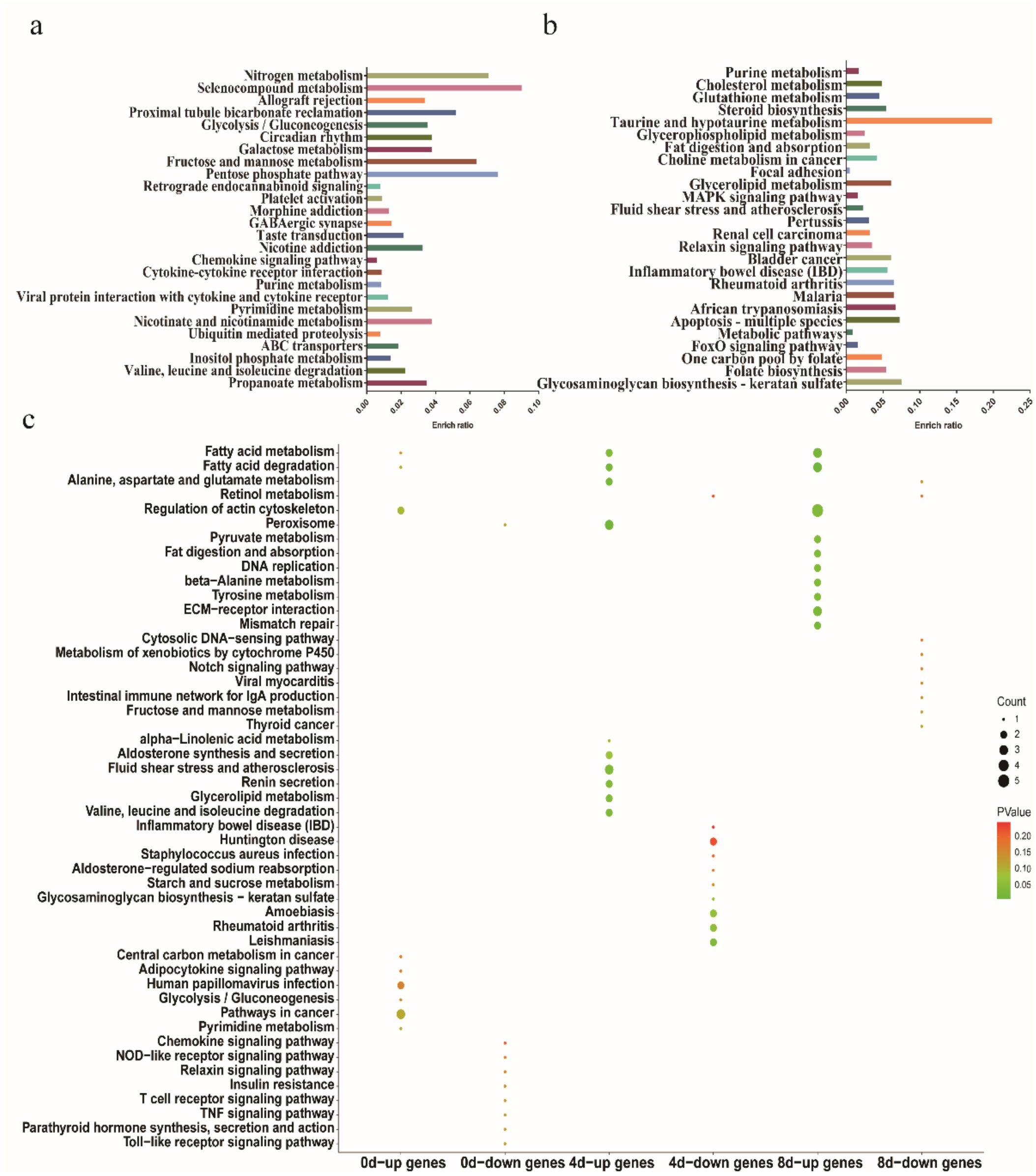

Figure S4. Kyoto Encyclopedia of Genes and Genomes (KEGG) analysis of target genes. (**a**) KEGG pathway analysis of differentially expressed miRNAs (DE-miRNAs) target genes from the adjacent differentiation time of subcutaneous adipocytes. (**b**) KEGG pathway analysis of DE-miRNAs target genes from the adjacent differentiation time of intramuscular adipocytes. (**c**) Pathway enrichment of DE-miRNAs target genes in the four stages in the BF vs IMF.

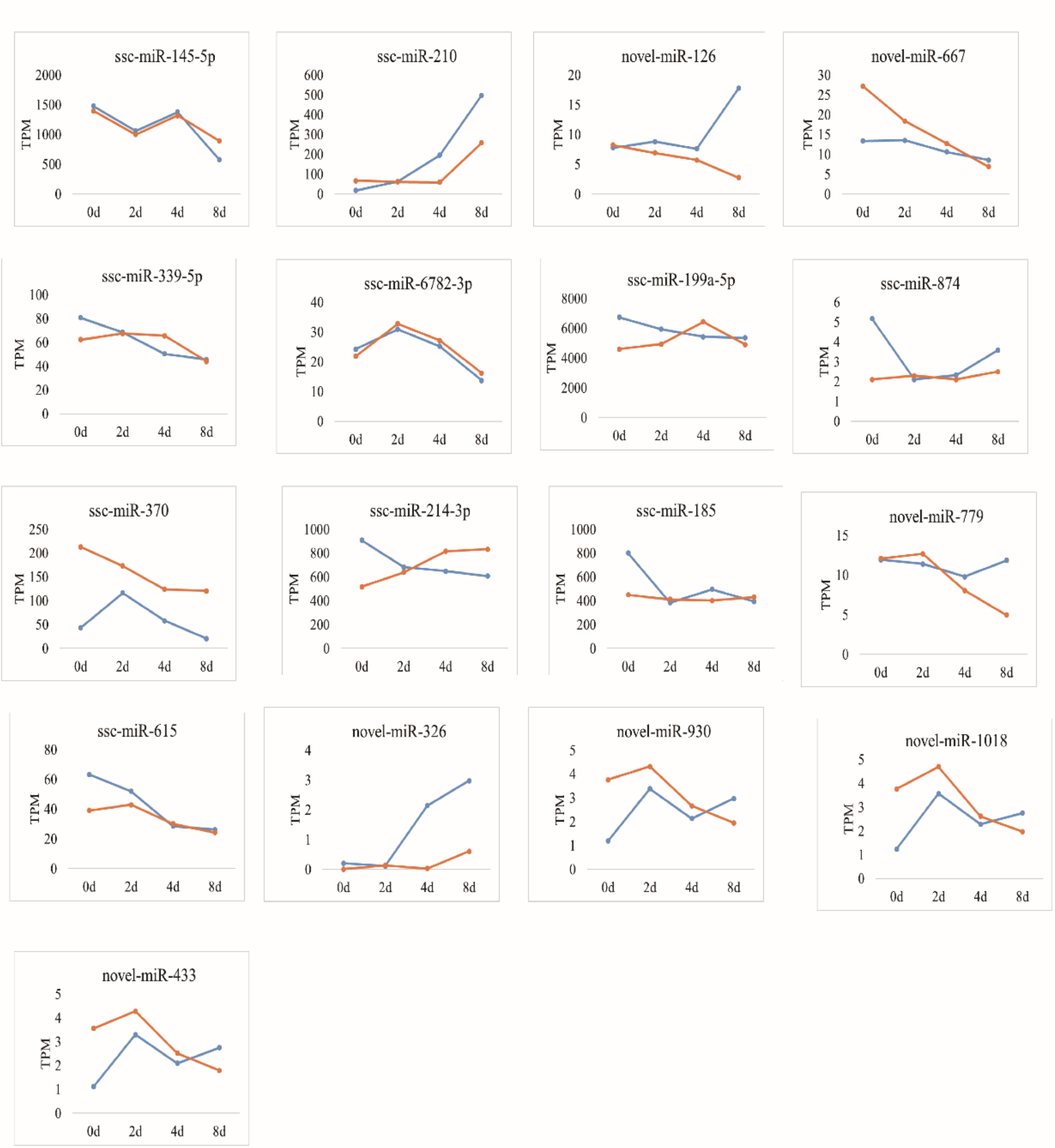

Figure S5. The expression of 17 key miRNA during the differentiation of two kinds of adipocytes. The orange broken line indicates the expression of the miRNA in subcutaneous adipocytes, and the blue indicates the expression of intramuscular adipocytes.
